# Supplementary material for: Expression in Pichia pastoris of Thermostable Endo-1,4-β-xylanase from the Actinobacterium Nocardiopsis halotolerans: Properties and Use for Saccharification of Xylan-Containing Products
Source: Int J Mol Sci. 2024 Aug 22;25(16):9121. doi: 10.3390/ijms25169121 (PMC11355003; doi:10.3390/ijms25169121)
Supplement: Supplementary file 1 [file ijms-25-09121-s001.zip › ijms-3105386-supplementary.pdf]

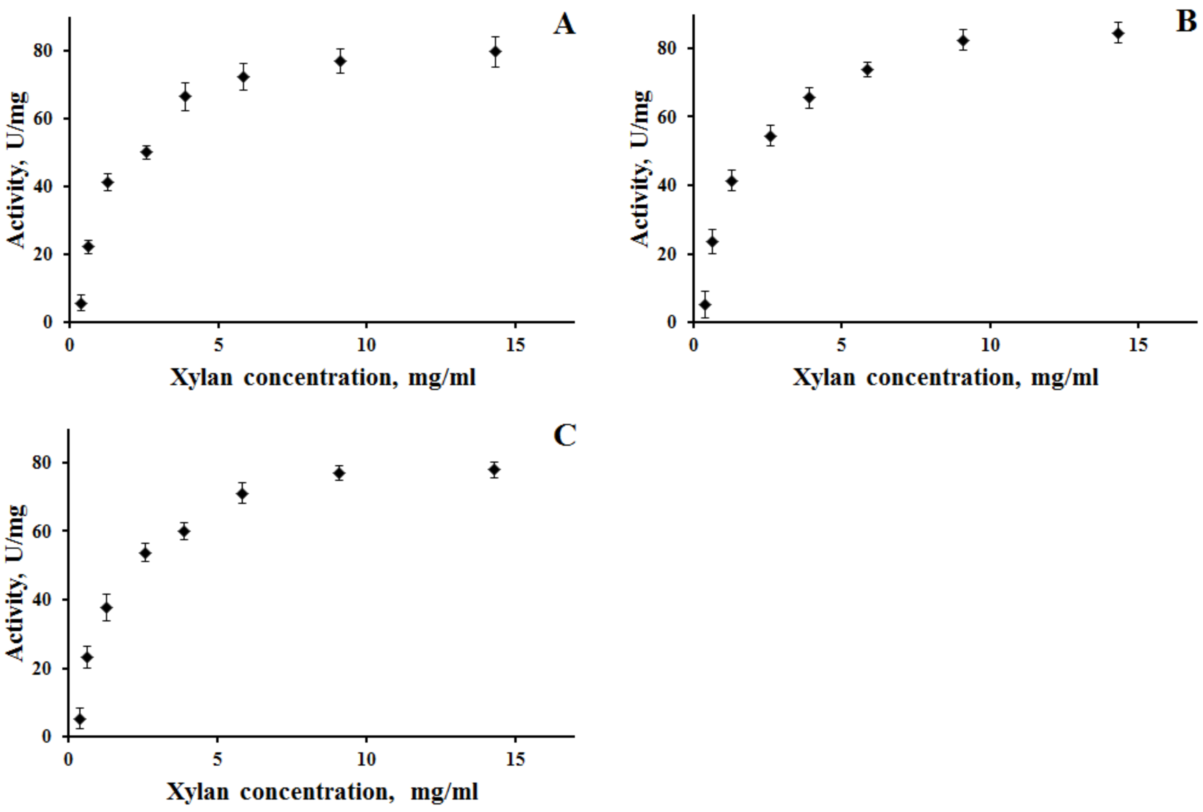

Figure S1. The dependence of NhX1 activity on xylan concentration. A, B, C - results of three independent experiments.
